# Emerging Nanopharmaceuticals and Nanonutraceuticals in Cancer Management

**DOI:** 10.3390/biomedicines8090347

**Published:** 2020-09-12

**Authors:** Lavinia Salama, Elizabeth R. Pastor, Tyler Stone, Shaker A. Mousa

**Affiliations:** The Pharmaceutical Research Institute, Albany College of Pharmacy and Health Sciences, Rensselaer, NY 12144, USA; lavinia.salama@gmail.com (L.S.); elizabeth.pastor@alumni.acphs.edu (E.R.P.); tyler.stone@acphs.edu (T.S.)

**Keywords:** dendrimers, liposomes, nanoparticles, nanopharmaceuticals, nanonutraceuticals, nanosuspension, polymeric micelles, quantum dots, solid lipid nanoparticles

## Abstract

Nanotechnology is the science of nanoscale, which is the scale of nanometers or one billionth of a meter. Nanotechnology encompasses a broad range of technologies, materials, and manufacturing processes that are used to design and/or enhance many products, including medicinal products. This technology has achieved considerable progress in the oncology field in recent years. Most chemotherapeutic agents are not specific to the cancer cells they are intended to treat, and they can harm healthy cells, leading to numerous adverse effects. Due to this non-specific targeting, it is not feasible to administer high doses that may harm healthy cells. Moreover, low doses can cause cancer cells to acquire resistance, thus making them hard to kill. A solution that could potentially enhance drug targeting and delivery lies in understanding the complexity of nanotechnology. Engineering pharmaceutical and natural products into nano-products can enhance the diagnosis and treatment of cancer. Novel nano-formulations such as liposomes, polymeric micelles, dendrimers, quantum dots, nano-suspensions, and gold nanoparticles have been shown to enhance the delivery of drugs. Improved delivery of chemotherapeutic agents targets cancer cells rather than healthy cells, thereby preventing undesirable side effects and decreasing chemotherapeutic drug resistance. Nanotechnology has also revolutionized cancer diagnosis by using nanotechnology-based imaging contrast agents that can specifically target and therefore enhance tumor detection. In addition to the delivery of drugs, nanotechnology can be used to deliver nutraceuticals like phytochemicals that have multiple properties, such as antioxidant activity, that protect cells from oxidative damage and reduce the risk of cancer. There have been multiple advancements and implications for the use of nanotechnology to enhance the delivery of both pharmaceutical and nutraceutical products in cancer prevention, diagnosis, and treatment.

## 1. Introduction

The evolution of nanoscale sciences and nanotechnology over the past two decades is responsible for the availability and growing prominence of the field of nanomedicine. Nanomedicine is a broad term to describe the use of devices or materials on the nanoscale (sizes in nm) to assess, diagnose, treat, or preserve health [1]. Applying nanomedicine could include modifying how drugs are delivered in the body as a means for optimization, using imaging for various purposes, and drug tracking within the body [1,2].

With the inception of nanomedicine, the community discovered the intrinsic ability of this science to circumvent some of the most fundamental shortcomings of traditional medicine, including off-target drug side effects, non-specific cell-targeting, and drug instability. Stemming from these advancements are the rapidly emerging novel formulations called nanopharmaceuticals and nanonutraceuticals [2]. Nanomedicine is at the forefront of anticancer medicine, owing to the potential to limit the aforementioned shortcomings of traditional medicine. Resistance to chemotherapy has been a major challenge in treatment, due to insufficient targeting and the presence of efflux pumps (p-glycoproteins (P-gp)) in the tumor that pumps out the chemotherapeutic agents. Nanoparticles (NPs) carry medications and have the ability to overcome resistance through specific targeting of tumor cells [3]. It is important to investigate the history and current knowledge of nanopharmaceuticals and nanonutraceuticals as a means to better understand the anticancer properties that these formulations hold and their potential in moving forward.

This review covers the current repertoire of nanopharmaceuticals and nanonutraceuticals that have been approved for cancer treatment or that exhibit extensively documented anticancer properties. We also give a brief summary of common nanoformulation techniques, their application potentials and limitations, as well as some novel developments in nanoscale formulations that show promise for future growth of nanopharmaceuticals and nanonutraceuticals.

## 2. Nanoformulations

The following nanoformulations are currently being widely studied in the medical community. Each one has specific advantages and disadvantages that make them unique. They all have applicability for use in cancer treatment or diagnosis. Some nano structures are better suited for certain types of drug delivery or tumor detection over others. Additionally, each of these existing drug delivery systems have paved the way for novel nanoformulations that have come to the forefront of studies in recent years.

Various nanoplatforms are being used, from liposomes over the past two decades to metallic NPs (iron and gold) in diagnosis to polymeric and solid NPs in targeted therapeutics, as highlighted below.

### 2.1. Liposomes

Conventional liposomes are spherical amphiphilic phospholipid vesicles, approximately 25 nm–2500 nm in size that can protect hydrophobic or hydrophilic materials from aqueous or non-aqueous environments, respectively, by forming a closed bilayer around the material [4]. This particular feature is beneficial for the delivery of both hydrophobic and hydrophilic chemotherapy to targeted sites. For mechanisms of delivery and composition, there are five classes of liposomes that can be utilized to optimize a product’s likelihood of achieving intended effects. These classes are pH-sensitive, cationic, conventional, long-circulating, and immuno-liposomes [2,5,6]. pH-sensitive liposomes stabilize when the external pH is altered, usually from a slightly alkaline or neutral pH to an acidic pH, which makes them stable at the physiological pH of 7.4 [7]. However, the pH-sensitive liposomes dissociate and release their content within the tumor, infected, or inflamed areas, which exhibit acidic properties [7]. Cationic liposomes are made using positively charged lipids and can interact with negatively-charged compounds in the body like DNA [8]. They can be used for the delivery of vaccines against cancer by loading synthetically long peptides into the liposomes to be delivered to dendritic cells, hence enhancing immune response [8]. Conventional liposomes were the first generation of liposomes and consist of a lipid bilayer. The bilayer can be neutral, cationic, or anionic phospholipids as well as cholesterol, which encompasses the aqueous volume [9]. The major disadvantage of conventional liposomes is their fast elimination from the blood. Long-circulating liposomes are generated by coupling of biocompatible polymers like polyethylene glycol (PEG) to the liposomal surface that protects the liposome from being cleared rapidly from the body [9]. Immuno-liposomes are created by attaching antibodies to the surface of the liposome. Such alteration in the liposomal structure allows for specific tissue targeting by binding to receptors specific to tumor cells [10].

Liposomal formulation is limited by its instability, poor solubility, high cost of production, and potential leakage of contained drug [4], though with recent advancements these issues are improving dramatically [11]. Liposomes have proven to be an efficacious and safe drug delivery system that is both biocompatible and biodegradable and does not have a risk of immunogenicity or toxicity [12]. They serve as a scaffold for carrying drugs and imaging agents, thereby increasing the circulation half-life and achieve targeting specificity. Doxorubicin HCL liposome (Doxil) is an example of a successful liposomal nano-anticancer agent used in the treatment of ovarian cancer, multiple myeloma, and AIDS-related Kaposi’s sarcoma [13,14]. Doxorubicin is a hydrophilic drug that is encapsulated inside the aqueous core of a liposome. The utilization of nanomedicine to deliver doxorubicin not only prolongs the half-life and increases the concentration of doxorubicin in tumor cells, but also leads to significant reduction in adverse events like cardiotoxicity because doxorubicin as Doxil is not bioavailable to cardiac cells [13].

Liposomes can also be used for imaging and have shown potential as contrast agents for magnetic resonance imaging (MRI) in in vivo studies [15]. Liposomes can serve as a vessel to deliver MRI paramagnetic contrast agents, such as gadolinium, thus increasing contrast specificity to parts of the body that are of interest and thereby reducing systemic toxicity [16].

### 2.2. Polymeric Micelles

Polymeric micelles (PMs) are amphiphilic block copolymers with an average diameter of 10–100 nm that arrange via self-aggregation into nanoscale core-shell structures [17]. The core consists of a dense hydrophobic region and the shell consists of hydrophilic co-polymers. PMs vary in their stability in blood and in drug release rate, and they may be tweaked by the choice of chemical linkage to their surface, e.g., esters [18]. The most widely studied copolymers are poly(ester)s, poly(L-amino acid)s, and poly(propylene oxide) [19]. Having a hydrophilic surface protects them from non-specific uptake, which allows this formulation to be used for systemic delivery of hydrophobic chemotherapeutic agents [20]. Another attractive quality of PMs owing to their hydrophilic shell and nanoscopic size is that mechanical clearance of the micelles by renal filtration, reticuloendothelial system (RES) uptake, and/or by the spleen is prevented, allowing for prolonged circulation in the blood [21]. An example of a PM-formulated anticancer drug is Genexol-PM, a PM formulation of paclitaxel [22]. Paclitaxel is a poorly water-soluble chemotherapeutic agent used in the treatment of ovarian cancer, breast cancer, non-small cell lung cancer, and AIDS-related Kaposi’s sarcomas [23]. In order to solubilize paclitaxel, it is often formulated in Cremophor EL (polyoxyethylated castor oil), which can lead to hypersensitivity reactions and requires pre-medication with diphenhydramine, corticosteroids, and H2 antagonists [23]. Genexol-PM overcomes the hypersensitivity reaction and has been approved in Korea for the treatment of advanced lung cancer and metastatic breast cancer [22].

### 2.3. Dendrimers

Dendrimers are tree-like branched-structure polymers whose shape and size are easily influenced and controlled via polymerization reactions. Branched nanostructures form around a spherical core with the ends of the dendrimers available for conjugation and molecule attachments [24,25]. Utilizing this structure permits drug and gene delivery (i.e., DNA/RNA) specificity as well as size and weight of molecule specificity, with exceptional entrapment efficiency. The high level of architectural control over dendrimers’ structure makes them compelling in various settings, including chemotherapy with reduced cytotoxicity, gene therapy, immune system stimulation, increasing bioavailability, and as contrast agents for MRI [25]. Like liposomes, dendrimers can increase the solubility and bioavailability of water-insoluble agents by either encapsulating the drug/oligonucleotide in their internal cavities or attaching them though electrostatic or hydrophobic interactions to their surface [26]. Dendrimers can also serve as vehicles to transport nucleic acid-based chemotherapies, which are hydrophilic molecules that cannot easily penetrate the cell membrane [26].

### 2.4. Quantum Dots

Quantum dots (QDs) are semiconducting materials in nanocrystal form, 2–10 nm in size, with an inorganic core and an organic-coated shell [27,28]. QDs can emit fluorescence when stimulated by light. This unique characteristic of QDs makes them a useful technology in imaging and tracking of intracellular processes [29,30]. QDs can accumulate in tumor tissue, allowing for tumor visualization and non-invasive diagnosis [30]. QDs have shown promising results when studied in vitro for rapid localizations of HER-2 receptors and for targeted chemotherapy and imaging-guided therapy [31].

### 2.5. Additional Novel Formulations

Adding to the commonly used formulations discussed above, Table 1 lists additional novel formulations, their size, and a brief summary of their characteristics and application potential.

## 3. Nanopharmaceuticals

The creation of nanotechnology birthed limitless potential in many fields. Most relevant for this review is the field of nanomedicine. Specifically, in cancer therapy this technology has allowed a choice of formulations with benefits that were previously unavailable. These benefits include targeting tumor cells, initiation of apoptosis, and accumulation of drug in specific tissue for increased exposure to cancer cells. Formulation of existing pharmaceuticals into the nanoscale has decreased toxicity and increased cell specificity. Table 2 summarizes nanopharmaceuticals that have been approved for the indicated applications. The success of these nanopharmaceuticals has proven the legitimacy of this technology and its potential in the field of oncology, a field that has been particularly limited by many obstacles, such as costs of development, regulatory frameworks, applicability of test protocols, and mechanisms to test for safety/efficacy [83]. As nanomedicine has matured, there has been vast commercialization of related technology, including hardware and software that can be used to create and customize these nanoformulations with reproducible quality and quantity in modest-sized lab settings [84].

Active nano-targeting for tumor-targeted delivery could be achieved using high-affinity ligand for a unique target that is overexpressed by cancer cells and their associated microenvironment, such as the case with integrin αvβ3, PSMA, or CD44 for delivery of chemotherapy into specific tumors [85,86,87,88,89,90,91]. Nanotechnology has been studied in vitro and in vivo to potentially restrict the action of the anti-angiogenic agent, diamino tetraiodothyroacetic acid (DAT) to the integrin avβ3 by conjugating DAT to PLGA NPs, forming DAT-conjugated PLGA (NDAT). The results of the study showed that nanotechnology allows for more specific tumor targeting, thereby allowing the use of lower doses of the toxic chemotherapeutic regimens [85]. Similarly, theranostic nanocarriers (folate-HBPE (CT20p)) were studied in the delivery of therapeutic peptide (CT20p) to allow for a more selective toxicity to prostate cancer cells that express PSMA. The results of the in vivo study indicated that nanocarriers can significantly and selectively facilitate tumor reduction [86]. Similar promising results were also shown in other studies, for example in the use of docetaxel nano-targeted delivery for the treatment of prostate cancer, use of NDAT for the targeted delivery of paclitaxel, cisplatin, and doxorubicin in tumor xenografts and in the delivery of toremifene in prostate cancer [87,88,89,90,91].

## 4. Nanonutraceuticals

Nutraceuticals are typically defined as pharmaceutical-grade standardized nutrients derived from food sources that, in addition to providing basic nutrients, also provide extra health benefits [92]. They mainly differ from non-pharmaceuticals in that they do not provide a pharmacologically active ingredient and instead deliver nutritional supplementation for medicinal purposes [93]. There has been widespread growth in the use of phytochemicals in nutraceuticals [94].

Phytochemicals are the naturally occurring chemicals in many plants that exhibit biologically active characteristics, including plant growth or defense. Many of these phytochemicals have been studied for centuries for their wide-ranging effects and medicinal value [94]. Phytochemicals like flavonoids suppress oxidative stress-induced DNA damage, primarily through their antioxidant properties, and thus they play a role in cancer chemoprevention [95,96]. Another example is the use of indoles found in cabbage that reduce the effects of estrogen and hence reduce the risk of developing breast cancer [97]. Capsaicin, found in chili pepper, is believed to protect DNA from carcinogens [98]. Formulating these nutraceuticals on the nanoscale, similarly to nanopharmaceuticals, may allow more pronounced and targeted effects that were previously unachievable.

Nutraceuticals derived from phytochemicals that have been studied for their potential anticancer properties exhibit promising results. Table 3 is a comprehensive list of phytochemicals that have been nano-formulated and studied in the corresponding cancer models. Moving forward, this subgroup of nanomedicine has great potential for growth because the major limiting characteristics of nutraceuticals, such as poor bioavailability and limited absorption in the gastrointestinal tract, can be overcome [99]. As the trend of seeking natural alternatives for prescription medications continues to grow, the younger, less-explored field of nanonutraceuticals may prove to be the lowest hanging fruit of all.

The physicochemical properties of phytochemicals such as polyphenols, phytosterols, carotenoids, vitamins, and minerals have a number of limitations, such as solubility, stability, and permeability, which could be improved using biodegradable nano assembly systems such as PLGA, chitosan, and natural fatty acids. Nanoformulation of natural bioactive compounds or natural extracts has demonstrated improved bio-accessibility/bioavailability. Different nanonutraceuticals demonstrated improved pharmacokinetic and pharmacodynamic properties that could facilitate their transition into pharmaceuticals and nanopharmaceuticals [125,174,175,176,177,178].

## 5. The Promising Future for Oncology with Nanomedicine

There are innumerable limitations when it comes to oncologic therapy. Most often cited is the lack of targeting and cell specification that leads to exaggerated negative side effects. These side effects are typically detrimental to patients’ health and can have a significant impact on quality of life. Using nanotechnology with enhanced tumor targeting capabilities may significantly save patients from immense pain and suffering and will combat multi-drug resistance and enhance tumor killing [179].

Nanotechnology has much potential in that it could be used for cancer diagnosis, treatment, and potentially the development of vaccines. Nanotechnology allows for more sensitive diagnosis of cancer by targeting specific cancer biomarkers, such as exosomes, cancer-associated proteins, circulating tumor DNA, and tumor cells [180]. Nanotechnology has been extensively studied in laboratory settings, however clinical data are often lacking. There are multiple ongoing trials looking at the use of nanotechnology in increasing the sensitivity and specificity of cancer diagnosis. A phase 1 trial is currently evaluating the use of ultrasmall silica particle in brain tumor imaging in humans [181]. This is the first human trial evaluating silica NPs in brain cancers. Understanding the mechanisms of distribution and excretion of the silica NPs in humans would be beneficial in future targeted oncological therapies. Carbon NPs are being studied as lymph node tracers in colorectal cancers to assess whether carbon NPs can increase lymph node yield after surgery [182]. Ferumoxytol-iron oxide NPs magnetic resonance is being evaluated as a potential tool to gain insights about the spread of cancers [183]. Another study evaluated the use of nanosensors in the diagnosis of gastric diseases [184]. Nanochip technology is also being studied as a potential tool for monitoring treatment response and detection of relapse in patients with diffuse large B-cell lymphomas [185]. As clinical research continues, we will see nanotechnology flourish in the field of oncology to aid in diagnosis and treatment.

## 6. Challenges and Promises in the Advancement of Nanoproducts (Nanomedicine)

Challenges facing the delivery of NPs carrying anticancer drugs to solid tumors include tumor microenvironments, matrix barriers such as fibrosis, collagen, and other matrices, tumor heterogeneity with variable vasculature, and the poorly vascularized tumor core. Current research in NP-based tumor drug delivery is focused on “active” targeting of NPs to the target rather than the traditional “passive” targeting through the enhanced permeability and retention (EPR) system.

Nonpharmaceutical formulations have distinct challenges from conventional pharmaceutical products that make their development more challenging. Cost of production of nanopharmaceuticals poses an important challenge. Nanopharmaceuticals are highly complex molecules that require careful selection of shape, vehicles, inorganic materials, and optimization of pharmacokinetic parameters to meet therapeutic needs and proper storage. Large-scale production of nanotherapeutics require high-efficiency equipment, time, and space. The higher the complexity of the desired nonpharmaceutical, the higher the cost of production and cost of acquisition. The cost-effectiveness of developing nanopharmaceuticals/nanonutraceuticals must be taken into consideration [186,187]. To justify their cost of production, a carefully planned and well-designed manufacturing process is going to be essential [188]. If careful manufacturing planning is not completed, reliability of nanotechnology in cancer diagnosis and treatment may be compromised. For example, there are various factors that affect the NP detection signaling and thus the sensitivity and specificity of cancer detection [189]. Manufacturing might not be an issue for all approved products that were aimed at passive targeting, but it might represent a challenge for active nano-targeting where there is a need to chemically conjugate the targeting moiety (small molecule, antibody, or aptamer) to the nanoplatform and then load the drugs to be delivered into the tumor and its microenvironment. However, those challenges can also be overcome. An example of such success is the case with BIND-014, a PSMA-targeted ACCURIN containing docetaxel, in patients with chemotherapy-naive metastatic castration-resistant prostate cancer. BIND-014 was clinically active and well-tolerated and the study met its primary endpoint, with 71% of patients achieving relative progression-free survival of at least six months. Unfortunately, the success rate for phase 3 trials was a mere ~14%, with failures stemming from lack of efficacy [190,191].

Physiochemical properties of the NPs such as shape, size, surface charges, surface ligands, absorption, distribution, metabolization, and excretion play a role in potential toxicity in the human body. Moreover, long-term toxicity from prolonged NP exposure (e.g., from nano-based imaging and treatment) cannot be fully and quickly known from in vivo studies [188].

Another important challenge to consider is FDA regulation of nanopharmaceuticals/nanonutraceuticals. Currently, the FDA approach to reviewing nanopharmaceuticals is the same as products that do not contain nanomaterials. Complexity and diversity of nano formulations is increasing significantly and thus the regulatory structure set forth by the FDA seems inadequate. Challenges involving safety, efficacy, and proper labeling are likely to arise [188,192,193].

Despite these challenges, nano-delivery of approved or new novel anticancer mechanisms holds great promise in the improved management of cancer and other disorders [194,195,196,197,198]. Nanoparticle-based drug delivery improves efficacy, solubility of hydrophobic drugs, half-lives of unstable compounds and proteins, and allows controlled and targeted release of drugs at the tumor site and its microenvironment. Ultimately, the integration of various enabling technologies and cross-collaboration with multidisciplinary theoretical and experimental scientists across academia and the pharmaceutical industry will accelerate these developments from the bench to the bedside and eventually to the market. Effective and safe cancer management continues to be a major issue in achieving improved survival and quality of life, because most anticancer drugs are cytotoxic and have a narrow therapeutic index.

## 7. Moving Forward Toward the Adaption of Nanomedicine toward Precision Medicine

Several nanopharmaceutical products in cancer and non-cancer indications are progressing at various stages of clinical development with great promises to improve the efficacy and safety of existing or new and novel compounds [199]. Clearly nanomedicine has provided recent success in drug delivery and tumor targetability that will increase the adaption of nanoproducts in cancer and beyond in the upcoming 2020–2030 decade. Nano-targeted delivery of active pharmaceuticals or biopharmaceuticals into the tumor site and its microenvironment should improve efficacy and safety as well as bypass hepatic pharmacogenomic variability in drug metabolism, allowing for better precision medicines in cancer and beyond.

## 8. Conclusions

The once hypothetical visions of what nanotechnology had to offer nanomedicine, specifically in the subgroups of nanopharmaceuticals and nanonutraceuticals, are now becoming reality. With the extensive investments that have been put into nanomedicine over the past two decades, there have been groundbreaking discoveries that paved the way to the approvals of many nanopharmaceuticals, with many more currently in process. In recent years, billions of dollars have been fronted to create academic institutes dedicated to the study of nanotechnology, and thus scientists are flocking to the field, with many dedicated to the study of nanomedicine. As nanomedicine continues to prove its value and legitimacy, nanoformulations of pharmaceuticals and nutraceuticals should continue to grow prolifically. Oncology, in particular, has many obstacles to overcome, because current treatment options are less than ideal. Thus, the options for oncology therapeutics are just beginning to propagate as the gold rush to nanomedicine matures. It is, however, important to remember that although nanotechnology holds great potential, it may not be the answer to curing all cancers.

## Figures and Tables

**Table 1:** Novel nanoformulations and their characteristics or applications in oncological medicine.

Nanoformulation	Size (nm)	Characteristics/Applications	References
Carbon nanotubes	0.5–3 by 20–1000	Hexagonal networks of carbon that form tube-like structures. Unique size, geometry, and surface characteristics make these optimal drug carriers for chemotherapy. Studied in vitro as a potential drug delivery system for controlled release of methotrexate (MTX); the nano-formulated agent significantly improved the antitumor function of MTX.	[32,33,34,35,36]
Fullerenes	0.7–1	Carbon atoms arranged in a cage-like structure. Used in imaging, drug delivery, photosensitizing, and stimulating immune response. Gd-metallofullerenol shown to deplete breast cancer stem cells, block epithelial-to-mesenchymal transition and was non-toxic to healthy mammary epithelial cells. Their targeted activity inhibited both tumor initiation and metastasis.	[37,38,39]
Gold nanoparticles (AuNP)	1–150	Can be fashioned into different shapes and sizes (e.g., gold nanorods, nanospheres, nanoshells, nanostars, nanocages). Unique shapes and sizes make them compelling for delivery of genes/oligonucleotides, proteins, and drugs to specific sites of interest and for cancer diagnosis and targeted phototherapy. MTX, when conjugated to AuNPs, accumulates more in tumor cells and at a faster rate than free MTX. Doxorubicin uptake was enhanced via AuNP conjugation in multi-drug resistant MCF7/ADR breast cancer, enhancing toxicity and overcoming drug resistance. Unique shape of gold nanostar allows light absorption and provides high photon-to-heat conversion efficiency, making them a compelling therapeutic option in tumor cell ablation.	[40,41,42,43,44]
Polymer-based nanoparticles	10–1000	Biodegradable polymers that are biocompatible and can load both hydrophobic and hydrophilic agents. Low toxicity and are cheaply fabricated. Most commonly used polymer is polylactic-co-glycolic acid (PLGA) nanoparticle. Their architectural design may affect their physiochemical properties, such as efficiency of drug encapsulation, particle size, distribution, stability, and shape. Introducing target moieties like folic acid, biotin, antibodies, and peptides to their surface that are specifically recognized by tumor cell receptors enhances targeting of chemotherapy. Development of PLGA nanoparticles with the peptide Pluronic P85, which inhibits drug efflux pump, both enhances tumor suppression and overcomes drug resistance. PLGA has been studied with other anticancer agents like mitramycin, paclitaxel, daunorubicin, and doxorubicin to enhance tumor targeting.	[45,46,47,48,49]
Iron oxide nanoparticles (IONP)	1–100	Type of magnetic nanoparticles (MNPs) with characteristically large surface area, small particle size, superparamagnetism, and magnetic response. Applications in diagnosis and targeted drug delivery. Most common use has been as MRI contrast agent to aid early detection of cancer. Ferumoxil (GastroMARK) is an example of an IONP that enhances MRI of gastrointestinal lumen. One study suggested that IONP inhibits tumor growth via induction of pro-inflammatory macrophages, particularly in liver cells, which accumulate high concentrations of IV IONP. IONPs can provide chemotherapeutic and magnetic hyperthermia therapy; they act as chemotherapy drug nanocarriers while generating localized heat when exposed to alternating magnetic field, e.g., conjugation of doxorubicin with magnetic oxide nanoparticle led to a higher cell killing response as a result of the alternating magnetic field, leading to heat generation by hyperthermia and release of doxorubicin inside the tumor cell, thus showing promising results in brain cancer cells treatment.	[50,51,52,53,54,55,56]
Artificial exosomes	50–120	Similar to liposomes, composed of a lipid bilayer and can encapsulate both hydrophobic and hydrophilic drugs. Easily PEGylated to enhance circulation time of a drug in blood. Also engineered with various targeting ligands and many proteins (like tetraspanins) that provide specific organotropism. Shown to improve potency and treat multi-drug-resistance cancers. For example, paclitaxel-encapsulated exosomes were effective in vitro against human pancreatic cells compared to control formulation.	[57,58,59]
Albumin nanovectors	5–140	Biocompatible, safe, and cost-effective to fabricate and can deliver both hydrophobic and hydrophilic drugs and diagnostic agents. Abraxane is an albumin-bound nano-formulation of paclitaxel and approved for the treatment of metastatic breast cancer, locally advanced or metastatic lung cancer, and metastatic adenocarcinoma of the pancreas.	[60,61]
Virosomes	~150	Spherical unilamellar vesicles that contain viral envelopes exclusive of the viral genome that serve as drug carriers in experimental cancer therapies. Mostly used in vaccine development like influenza (Inflexal) and hepatitis A (Epaxal) vaccines. Phase 1 trial for using virosome to formulate Her-2/neu multi-peptide vaccine resulted in induction of anti-Her-2/neu 2 specific antibodies in patients with metastatic breast cancer.	[62,63]
Silica-based nanoparticles	20–200	Mesoporous silica-based nanoparticle (MSN) structures can be used to load and deliver antitumor agents. Well-ordered internal mesopores (~2–6 nm), large surface area, modifiable size, easy surface modifications, shape, and robustness make them ideal nano-delivery systems. Use of MSNs for doxorubicin delivery has improved the ability to cross the blood–brain barrier in cell models, thus making MSNs ideal for delivery of antitumor agents to the brain as in glioblastomas. Another example is formulation of cisplatin with silica-based nanoparticles for release into brain cancer cells.	[64,65,66,67,68,69]
Nanoshells	<100	Silica core coated with metallic outer shell. Properties modifiable by adjusting shell-to-core ratio. Used for diagnostic, therapy, immunologic. Can be contrast agents to image HER2 clinical marker in breast cancer. Nanoshells’ exposure to HER2 or IgG PEGylated antibodies facilitates targeting of breast cancer cells.	[70,71,72]
Nanobubbles	40–800	Bubble-like structures generated against hydrophobic surfaces in liquids. Cancer drugs can be incorporated and easily visualized via ultrasound. Internalization of drugs to tumor cells when nanobubble accumulates inside tumor’s interstitium due to their ability to extravasate through defective tumor microvasculature. Once inside, nanobubble acts as a strong contrast for ultrasound and once imaging is achieved, drug is released from nanobubble.	[73,74,75]
Niosomes	25–100	Non-ionic, self-associating surfactant-based vesicle in an aqueous phase. Unique delivery system for both hydrophilic and lipophilic drugs such as chemotherapies. Have potential for targeted delivery of chemotherapy to desired tumor site. Niosomal encapsulation of methotrexate (MTX) and doxorubicin has shown increased delivery to tumor and increased tumor killing.	[76,77,78]
Nanosuspensions	<1000	Fine colloid, solid pharmaceutically active ingredient particles suspended and dispersed in aqueous vehicles. Increases bioavailability and dissolution rate of a drug. Used in in vitro and in vivo studies to formulate injectable sorafenib for treatment of hepatocellular carcinoma. Sorafenib nanosuspension showed significantly superior antitumor effect when compared to oral and injectable sorafenib.	[79,80,81,82]

**Table 2:** Approved oncological nanopharmaceuticals and their applications.

Product	Drug	Formulation	Company	ROA	Application (Approval Year)
DaunoXome	Daunorubicin Citrate	Liposome	Galen	IV	Kaposi Sarcoma (1996)
DepoCyt	Cytarabine	Liposome	Pacira	IT	Neoplastic and Lymphomatous Meningitis (1999)
Doxil	Doxorubicin HCl	Liposome	Janssen	IV	Kaposi Sarcoma, Ovarian cancer, Multiple myeloma (1995)
Marqibo	Vincristine Sulfate	Liposome	Spectrum	IV	Acute Lymphoid Leukemia (2012)
Mepact	Mifamurtide	Liposome	Takeda	IV	High-grade Non-metastatic Osteosarcoma (EU 2009)
Myocet	Doxorubicin	Liposome	Teva	IV	Metastatic Breast Cancer (EU 2000)
Neulasta	Filgrastim	PEGylated protein	Amgen	SC	Febrile Neutropenia, in Non-myeloid Malignancies (2002)
Oncaspar	Pegaspargase	PEGylated protein	Shire	IM/IV	Acute Lymphoblastic Leukemia (1994)
Eligard	Leuprolide Acetate	Polymer-based	Tolmar	SC	Advanced Prostate Cancer (2002)
Genexol	Paclitaxel	Polymer-based	Samyang	IV	Pancreatic Cancer, Metastatic Breast Cancer (SK 2001)
Opaxio	Paclitaxel	Polymer-based	CTI Biopharma	IV	Glioblastoma, NSC Lung Cancer, Ovarian cancer (pending)
Zinostatin stimalamer	Styrenemaleic acid and NCS protein	Polymer-based	Astellas	IV	Hepatoma (JP 1994)
Abraxane	Albumin and paclitaxel	Protein-drug conj.	Celgene	IV	Metastatic Breast Cancer, NSC Lung Cancer, Metastatic Adenocarcinoma of the Pancreas (2005)
Kadcyla	Trastuzumab emtansine	Protein-drug conj.	Genentech	IV	Metastatic Breast Cancer (2013)
Ontak	Denileukin diftitox	Protein-drug conj.	Eisai	IV	Persistent or Recurrent Cutaneous T-cell Lymphoma (1999)
NanoTherm	Iron oxide+aminosilane	Metal-based	MagForce	ITU	Prostate cancer, Pancreatic cancer, Glioblastoma (EU 2013)
Gendicine	rAd-p53	Virosome	Shenzhen	ITU	Head and Neck Squamous Cell Carcinoma (CN 2003)
Rexin-G	Cyclin G1 inhibitor	Virosome	Epeius	IV	Solid Tumors (PH 2007)

ROA, route of administration; IV, intravenous; IT, intrathecal; SC, subcutaneous; IM, intramuscular; ITU, intratumorally; PEG, polyethylene glycol; Two letters before year notates approval in specific countries/regions; EU, Europe; SK, South Korea; JP, Japan; CN, Canada; PH, Philippines; all others approved in the United States.

**Table 3:** Phytochemicals and their application in nanomedicine.

Phytochemical	Application/Targets	Delivery System
**β-Lapachone**	Colon cancer cells, lung [100], prostate, breast cancer cells [101]	PEG-PLA polymer micelles [102,103]
**Curcumin**	Brain [104], leukemia, colon [105], breast [106], prostate [107], cervical [108,109,110], pancreatic cancer cells [111], neuroblastoma [112]	PLGA, PLA-vitamin E TPGS copolymer, alginate NPs, soy protein NPs, PVP conjugate micelle, α-CD derivatives, thermosensitive polymer NP, nanoprecipitation, liposomal formulation, magnetic NP, hollow capsules, albumin nanosuspension [113,114]
**Daidzein**	Cardiovascular system [115], breast cancer [116]	SLN with PEGylated phospholipid
**Dibenzoylmethane**	Cervical cancer cells, hepatic cancer, prostate cancer, lung cancer, osteosarcoma [117,118,119]	PLA NP
**Dihydroartemisinin**	Breast cancer [120], ovarian cancer [121], esophageal cancer [122]	Magnetic NP
**Ellagic acid**	Breast cancer [123], prostate cancer, colorectal cancer, melanoma, ovarian cancer, non-small cell lung cancer (NSCLC), bladder cancer [124,125]	PLGA NP, PEG, mesoporous silica NP
**Epigallocatechin Gallate (EGCG)**	Prostate cancer cells, pancreatic cancer cells [126], breast cancer [127], ovarian cancer, endometrial cancer, renal cancer, and colon cancer	Lipid NP, polymeric NP (PLA and PLGA), liposomes, gold NP, selenium nanocarriers, PEG [128]
**Eugenol**	Colon and liver cancer [129], breast cancer [130,131]	Nanoemulsions, magnetic NP
**Ferulic acid**	Hepatocellular cancer, colon cancer [132], pancreatic cancer [133]	NP, chitosan-coated SLN
**Gambogic acid**	Breast cancer, pancreatic cancer [134]	Glycol chitosan NP, NP coated with red blood cell membranes [135], carbo nanotube and graphene nanodelivery, magnetic NPs
**Genistein**	Breast cancer, prostate cancer, colon cancer [136], osteosarcoma, human gastric carcinoma, neuroblastoma, bladder cancer, lung cancer, cervical cancer [137]	Biodegradable TPGS-b-PCL NP, PEGylated silica NP, hybrid nanomaterial
**Honokiol**	Liver cancer [138], breast cancer [139], colorectal cancer [140], lymphoid malignant cells [141], melanoma [142], thyroid cancer [143], glioblastoma [144]	NP, nanosome, QD
**Naringenin**	Cervical cancer [145], glioblastoma, lung cancer [146], pancreatic cancer [147]	Silk fibroin NP, naringenin loaded PCL NP, multi-walled carbon nanotubes, naringenin-loaded PLGA NP
**Nobiletin**	Breast cancer, ovarian cancer, prostate cancer, colon cancer [148], liver cancer, hepatocellular carcinomas, glioblastoma, gastric cancer, lung cancer, nasopharyngeal cancer [149]	Nano-emulsion [150], micelles
**Quercetin**	Breast cancer [151], ovarian cancer [152,153], cervical cancer, prostate cancer, colorectal cancer, gastrointestinal cancer, liver cancer [154], thyroid cancer, lung cancer, pancreatic cancer, lymphomas	Quercetin encapsulated in SLN [155], gold NP-quercetin into PLGA, graphene oxide nanocarrier
**Resveratrol**	Skin cancer, breast cancer, prostate cancer, pancreatic cancer	Gold NP [156], SLN [157]
**Thymoquinone**	Glioblastoma [158], breast cancer, ovarian cancer, osteosarcoma, colorectal cancer, adenocarcinoma, pancreatic carcinoma, myeloblastic leukemia [159,160]	Silica NP core loaded with thymoquinone, thymoquinone-loaded nanostructured lipid carriers, PLGA and PEG, thymoquinone-encapsulated chitosan NP, 1,2-dipalmitoyl-sn-glycerol-3-phosphocholine liposomal system, micelles
**Triptolide**	Breast cancer [161], liver cancer, lung cancer, pancreatic cancer [162,163]	Triptolide-loaded cationic liposomes, triptolide coupled to vitamin E using dithiodiglycolic acid and co-dissolved with PEG2000-linoleic acid, nucleolin-specific aptamer mediated polymeric nanocarrier
**Taxifolin**	Colorectal cancer, breast cancer [164] [165,166]	Zinc oxide NP, NP by liquid antisolvent precipitation
**Ursolic acid**	Cervical cancer [167], breast cancer [168], prostate cancer, lung cancer, hepatocellular carcinoma [169], gall bladder carcinoma, melanoma [170]	Gold-ursolic acid into PLGA NP, long-circulating and pH-sensitive liposomes [171], PEG modified liposome [172], ursolic acid-loaded chitosan NP [173], pH-sensitive mesoporous silica NP

CD, cyclodextrin; NP, nanoparticle; PCL, polycaprolactone; PEG, polyethylene glycol; PLA, poly(lactide); PLGA, poly lactic-co-glycolic acid; PVP, poly(vinyl pyrrolidone); QD, quantum dot; SLN, solid lipid NP; TPGS, tocopheryl polyethylene glycol 1000 succinate.
